# Fibrinogen levels in trauma patients during the first seven days after fibrinogen concentrate therapy: a retrospective study

**DOI:** 10.1186/s13049-016-0221-8

**Published:** 2016-03-12

**Authors:** Christoph J. Schlimp, Martin Ponschab, Wolfgang Voelckel, Benjamin Treichl, Marc Maegele, Herbert Schöchl

**Affiliations:** Ludwig Boltzmann Institute for Experimental and Clinical Traumatology, AUVA Research Centre, Vienna, Austria; Department of Anaesthesiology and Intensive Care, AUVA Trauma Centre, Academic Teaching Hospital of the Paracelsus Medical University, Franz Rehrl Platz 5, 5020 Salzburg, Austria; Department of Anaesthesiology and Intensive Care, Innsbruck Medical University, Innsbruck, Austria; Department of Traumatology, Orthopedic Surgery and Sports Medicine Cologne-Merheim Medical Center (CMMC), Institute for Research in Operative Medicine (IFOM), Cologne, Germany

**Keywords:** Blood coagulation tests, Fibrinogen, Plasma, Trauma

## Abstract

**Background:**

Fibrinogen concentrate (FC) is increasingly used as first line therapy in bleeding trauma patients. It remains unproven whether FC application increases post-traumatic plasma fibrinogen concentration (FIB) in injured patients, possibly constituting a prothrombotic risk. Thus, we investigated the evolution of FIB following trauma in patients with or without FC therapy.

**Methods:**

At the AUVA Trauma Centre, Salzburg, we performed a retrospective study of patients admitted to the emergency room and whose FIB levels were documented thereafter up to day 7 post-trauma. Patients were categorized into those with (treatment group) or without (control group) FC therapy during the first 24 h after hospital admission. A subgroup analysis was carried out to investigate the influence of the amount of FC given.

**Results:**

The study enrolled 435 patients: treatment group, *n* = 242 (56 %); control group, *n* = 193 (44 %), with median Injury Severity Score of 34 vs. 22 (*P* < 0.001) and massive transfusion rate of 18.4 % vs. 0.2 % (*P* < 0.001). In the treatment group (median FC dose 6 g), FIB was lower on admission and up to day 2 compared with the control group. In patients receiving high (≥10 g) doses of FC, FIB was lower up to day 5 as compared to controls. At other timepoints, FIB did not differ significantly between the groups. In the treatment vs. the control group, other coagulation parameters such as prothrombin time index and platelet count were consistently lower, while activated partial thromboplastin time was consistently prolonged at most timepoints. Inflammatory parameters such as C-reactive protein, interleukin-6 and procalcitonin were generally lower in controls.

**Discussion:**

The rise of FIB levels from day 2 onwards in our study can be attributed to an upregulated fibrinogen synthesis in the liver, occurring in both study groups as part of the acute phase response after tissue injury.

**Conclusions:**

The treatment of severe trauma patients with FC during bleeding management in the first 24 h after hospital admission does not lead to higher FIB levels post-trauma beyond that occurring naturally due to the acute phase response.

## Background

Acquired fibrinogen deficiency is known to occur in trauma [1]; plasma fibrinogen (FIB) levels on admission to the emergency room (ER) are strongly correlated with dilution, shock and the severity of injury [2, 3]. In bleeding patients, FIB reaches critically low levels earlier than any other coagulation factors [4], and low FIB levels have been associated with increased blood loss and/or transfusion requirements in a number of settings, including trauma [5, 6], cardiac surgery [7, 8] and post-partum haemorrhage [9]. During major traumatic haemorrhage, FIB is almost always the primary coagulation factor deficiency [10] and low FIB has been shown to be a strong independent risk factor for death in trauma patients requiring massive transfusion [3, 11, 12]. Early replacement of fibrinogen appears to be beneficial in trauma patients experiencing severe bleeding [3, 13–16]. The recent European trauma and perioperative bleeding guidelines recommend a FIB threshold concentration of 1.5–2.0 g/l as a trigger for replacement therapy in bleeding patients [17, 18].

Besides using antifibrinolytics when necessary, some trauma centres employing goal-directed haemostatic therapy algorithms use fibrinogen concentrate (FC) as the primary pharmacological intervention in trauma-induced coagulopathy [19–21]. It has been questioned whether the use of FC may lead to large, sustained increases in FIB levels, thus possibly constituting a prothrombotic risk [22].

However, few data exist on the time course of the haemostatic effects of FC use in clinical practice. In aortic surgery, it has been shown that no differences exist in the evolution of FIB on postoperative day 2 and day 10 between patients receiving either FC or fresh frozen plasma (FFP), or both, during surgery [23]. In trauma, there are only few published data on the time course of FIB beyond 24 h post-injury in patients who have received FC [20, 24]. Recently it was shown in a small prospective study on 77 trauma patients receiving FC ± prothrombin complex concentrate (PCC) or without any coagulation factor concentrate therapy that FIB levels increase in a similar pattern over 7 days [25].

In the current retrospective study, we primarily aimed to evaluate FIB levels on seven consecutive days following traumatic injury in a large population of patients with or without FC therapy and furthermore analysed whether the amount of FC given in the first 24 h influences the time course of FIB over 7 days. Secondly, we evaluated the corresponding time course of other coagulation, haematological and inflammatory parameters.

## Methods

The Ethics Committee of the federal state of Salzburg (Ethikkommission für das Bundesland Salzburg) approved (Protocol: 415-EP/73/197-2013) this retrospective analysis of data from patients admitted to the ER of the AUVA Trauma Centre, Salzburg, Austria, between January 2005 and June 2015. The need for informed consent was waived.

Severely injured patients in our institution are generally treated with a standardised goal-directed haemostatic therapy algorithm using thromboelastometry (ROTEM®) [26], and are admitted to the intensive care unit (ICU) using standardised treatment protocols for trauma patients. The main inclusion criterion for a patient’s dataset was the recording of FIB levels in our hospital trauma database from admission to the ER up to day 7 post-trauma (any type, including traumatic brain injury). Other inclusion criteria were: available documentation of Injury Severity Score (ISS), hospital mortality, sex and age; receipt and amount of allogeneic blood products (e.g., red blood cells [RBC], FFP and platelet concentrates) and coagulation factor concentrates (e.g., FC and PCC) in the first 24 h. Patients were excluded from the study if they did not have available fibrinogen data on day 6 or 7 post-trauma or if they died within the first 7 days. Datasets from patients already participating in another 7 day follow-up study were also excluded from this study [25].

Blood samples for analysis were collected according to in-hospital standards at the following timepoints: on admission to the ER, and every morning thereafter up to day 7. Citrated blood samples were centrifuged and the following coagulation parameters were assessed: fibrinogen concentration (Clauss method with electro-mechanical detection using a STA-Compact analyser [Diagnostica Stago S.A.S., Asnières sur Seine, France]; normal range 2.0–4.5 g/l), prothrombin time index (PTI; measured using a Sysmex XE- 2100 [Roche Diagnostics, Mannheim, Germany]; expressed as a percentage ratio, normal range 70–120 %), activated partial thromboplastin time (aPTT; measured using Sysmex XE-2100; normal range 23.7–34.9 s) and antithrombin III (AT III; normal range 80–120 %). Haemoglobin, white blood cell count and platelet count were determined on a Sysmex SF-3000 (Sysmex Corporation, Kobe, Japan) and base excess was determined on a Roche OMNI® S Blood Gas Analyser (Roche Diagnostics, Mannheim, Germany). Interleukin 6 (IL-6), procalcitonin (PCT), and C-reactive protein (CRP) were determined either on a Cobas Integra or Cobas 6000 (both Roche Diagnostics, Mannheim, Germany).

All eligible datasets were primarily categorized into two groups: patients who received any amount of FC during the first 24 h after hospital admission (treatment group) and patients who did not receive any source of fibrinogen supplementation (control group). Secondly, for subgroup analyses the treatment group was subdivided into three categories according to the dose of FC administered (1–4 g, 5–9 g and ≥10 g).

### Statistical analysis

Continuous study variables were analysed for normal distribution by the Kolmogorov-Smirnov test. Based on the underlying distribution in the treatment and control groups, between-group comparisons were analysed using the Mann–Whitney *U* test. For between-group comparisons of categorical variables, Fisher’s exact test was used. Differences between four subgroups were analysed with ANOVA/Kruskal-Wallis and the Chi-Square test. For the subgroup analyses based on four different FC dose ranges (0 g, 1–4 g, 5–9 g and ≥10 g) at each timepoint, the Newman-Keuls multiple comparison test was used. Unless otherwise stated, data are presented as median (interquartile range [IQR]) for continuous variables, and as number (%) for categorical variables. For all statistical tests, a *P*-value <0.05 was considered significant. Statistical calculations were performed using GraphPad Prism 5 (GraphPad Software, La Jolla, CA, USA).

## Results

### Patient characteristics

A total of 435 patients with predominantly blunt trauma fulfilled the inclusion criteria for this study: 242 (56 %) in the treatment group and 193 (44 %) in the control group (FC = 0 g). Of the 435 patients, 363 (83 %) were male. Overall, the median (IQR) age was 45 years (28–59) and the median ISS was 27 (20–36), with 409 (94 %) patients classified as ISS ≥16. ISS was significantly higher in patients in the treatment group compared with the control group (34 [24–42] vs. 22 [17–27.5]; *P* < 0.0001). Age was significantly lower in the treatment vs. the control group (42 [25–54] years vs. 50 [37–65] years; *P* < 0.0001). Mortality was similar in both groups (17 patients in the treatment group [3.9 %] vs. 12 patients [2.8 %] in the control group; *P* = 0.85). The number of patients who required massive transfusion (≥10 RBC units in 24 h) was significantly higher in the treatment group than in the control group (79 [18.4 %] vs. 1 [0.2 %]; *P* < 0.0001). Overall, patients in the treatment group received 6 (3–11) units of RBCs vs. 0 (0–0) units in the control group in the first 24 h (*P* < 0.0001). During the same period a median of 6 g (4–9) FC was administered to patients in the treatment group only. Patient characteristics of the control group and each FC dose subgroup are shown in Table 1.

**Table 1:** Patient characteristics of the control group and each fibrinogen concentrate dose subgroup

	Control group	1–4 g FC	5–9 g FC	≥10 g FC	*P*-value^a^
	(*n* = 193)	(*n* = 97)	(*n* = 93)	(*n* = 52)	
Age, years	50 (37–65)	40 (22–56)	41 (26.5–53)	43.5 (28–53.75)	<0.0001
Male, *n* (%)	153 (79.3)	69 (71.1)	75 (80.6)	39 (75.0)	0.3513
ISS	22 (17–27.5)	29 (25–40)	34 (23–45)	41.5 (33.25–50)	<0.0001
Mortality, *n* (%)	12 (6.2)	4 (4.1)	5 (5.4)	8 (15.4)	0.0533
Massive transfusion, *n* (%)	1 (0.5)	8 (8.2)	26 (28.0)	45 (86.5)	<0.0001
FC, g	–	3 (2–4)	6 (6–8)	12 (10.25–15.75)	<0.0001
RBC					
units (total)	0 (0–0)	3 (2–6)	7 (5–10)	15 (11.35–20.75)	<0.0001
*n* (%)	25 (13.0)	74 (76.3)	87 (93.5)	52 (100)	<0.0001
units^b^	3 (2–4.5)	4.5 (3–7)	7.0 (5–10)	15.0 (11.25–20.75)	<0.0001
PCC					
IU (total)	0 (0–0)	0 (0–0)	300 (0–1800)	3600 (1800–5400)	<0.0001
*n* (%)	5 (2.6)	22 (22.7)	46 (49.5)	50 (96.2)	<0.0001
IU^b^	1800 (1200–4500)	1200 (1150–1800)	1800 (1425–2400)	3600 (1800–5425)	<0.0001
Platelet concentrate					
units (total)	–	0 (0–0)	0 (0–0)	1 (0–2)	<0.0001
*n* (%)	–	1 (1.0)	17 (18.2)	28 (53.8)	<0.0001
units^b^	–	2 (2–2)	2 (1.5–2.0)	2.0 (2.0–3.75)	0.2441
FFP					
units (total)	–	–	0 (0–0)	0 (0–0)	<0.0001
*n* (%)	–	–	3 (3.2)	10 (19.2)	<0.0001
units^b^	–	–	5 (5–8)	10 (10–13)	0.0097

*FC* fibrinogen concentrate, *FFP* fresh frozen plasma, *ISS* injury severity score, IU international units, *PCC* prothrombin complex concentrate, *RBC* red blood cells. ^a^ANOVA (Kruskal Wallis) *P* value for all four groups; ^b^Median (interquartile range) in patients who received that particular product

Data presented as median (interquartile range), number (%)

### Time course of laboratory parameters in the control and treatment groups

The time courses of FIB, PTI, aPTT, AT-III, platelet count, haemoglobin, WBC, CRP, IL-6 and PCT during the first 7 days after trauma are shown in Table 2.

**Table 2:** Time course of plasma fibrinogen, PTI, aPTT, AT-III, platelet count, haemoglobin, WBC, CRP, IL-6 and PCT during 7 days after trauma in overall treatment group and control group

	ER	Day 1	Day 2	Day 3	Day 4	Day 5	Day 6	Day 7
Fibrinogen (g/l)								
Treatment	1.58 (1.07–2.03)	2.33 (2.02–2.71)	4.15 (3.47–4.83)	5.51 (4.72–6.7)	6.12 (5.12–7.54)	6.55 (5.3–7.9)	6.8 (5.41–8.19)	6.99 (5.9–8.49)
*n*	242	242	238	239	231	227	224	207
Control	2.46 (2–2.98)	2.75 (2.25–3.25)	4.36 (3.77–5.32)	5.74 (4.74–6.74)	6.23 (5.07–7.64)	6.67 (5.25–7.9)	6.95 (5.56–8.24)	7 (5.59–8.45)
*n*	191	174	169	150	149	145	147	139
*P*-value	<0.0001	<0.0001	0.0034	0.2122	0.9996	0.6106	0.6995	0.6602
PTI (%)								
Treatment	66 (54–78.5)	60 (49–69)	60 (51–72)	73 (63–86)	82 (69–93)	84 (70–99)	87.5 (73–98)	87 (77–98)
*n*	241	242	238	238	231	227	224	207
Control	90 (78–101)	75 (64–85)	78 (64–87)	82 (70–94)	89 (76.5–99)	92 (79–101)	94 (84–104)	92 (81–103)
*n*	192	175	169	150	149	145	147	140
* P*-value	<0.0001	<0.0001	<0.0001	<0.0001	0.0009	0.0012	<0.0001	0.0038
aPTT (s)								
Treatment	30 (26.2–36.4)	39.5 (35.4–45.3)	39.7 (35.5–45.2)	38.3 (34.2–43.5)	36.1 (32–40.8)	34.3 (30.9–39.1)	33.2 (30–37.7)	32.3 (29.3–37.2)
*n*	237	238	234	236	231	227	224	208
Control	26.6 (24.1–28.9)	32.6 (30–36.6)	35 (31.6–38.3)	34.1 (31.5–38)	33 (29.7–36.3)	31.3 (28.9–35.5)	30.5 (28–34.4)	30 (27.4–33.3)
*n*	192	175	168	149	149	145	147	140
* P*-value	<0.0001	<0.0001	<0.0001	<0.0001	<0.0001	<0.0001	<0.0001	<0.0001
AT-III (%)								
Treatment	73 (61–86)	57 (46.3–66)	57 (46.8–68)	57.5 (48–68.8)	64 (53–75)	71 (60–86)	82 (64–96)	89.5 (75–103)
*n*	103	204	182	164	152	131	119	116
Control	88 (77–97)	74.5 (65.3–84)	75 (64–85)	76 (60.5–84)	85 (72–96.5)	90 (72.5–102.5)	98 (85–108.5)	100 (89.8–111.3)
*n*	115	132	99	78	73	85	82	70
* P*-value	<0.0001	<0.0001	<0.0001	<0.0001	<0.0001	<0.0001	<0.0001	0.0010
Platelet count (×10^9^/l)								
Treatment	184 (143–230)	96 (64.8–134.3)	91.5 (64.8–117)	92 (65.5–120)	111.5 (76–144)	134 (94–174)	160 (116.8–209)	205 (158–266)
*n*	241	242	242	241	238	237	238	229
Control	222 (174–262)	158 (130–189.5)	141 (109–175)	143 (109–175)	160 (130–200.5)	184 (155–224.8)	209 (171.5–250)	240 (197–295)
*n*	193	185	185	179	178	180	173	163
* P*-value	<0.0001	<0.0001	<0.0001	<0.0001	<0.0001	<0.0001	<0.0001	<0.0001
Haemoglobin (g/l)								
Treatment	110 (90–127)	90 (82–102)	90 (80–98)	88 (80–97)	90 (81–98)	90 (82–98)	91 (84–99)	90 (81–97)
*n*	242	242	242	242	241	238	238	232
Control	132 (118–142)	100 (90–111)	93 (84–109)	92 (82–106)	93 (84–104)	94 (85–105)	95 (86–106)	94 (87–106)
*n*	193	187	190	184	184	185	178	166
* P*-value	<0.0001	<0.0001	0.0004	0.0008	0.0103	0.0005	0.0013	<0.0001
White Blood Cell count (×10^9^/l)								
Treatment	13.1 (10–17.7)	7.1 (5.5–9.4)	8.6 (6.5–10.4)	7.9 (6.1–9.9)	7.4 (5.7–9.9)	7.9 (6.1–10.3)	9 (7.3–11.4)	10.2 (8–12.9)
*n*	178	203	205	203	201	201	204	202
Control	13.3 (10.8–17.3)	8.2 (6.4–9.9)	8.1 (6.7–9.9)	7.3 (6–9.6)	7.2 (5.6–9.3)	8 (6.3–9.9)	9.3 (7–11.1)	9.6 (7.3–11.7)
*n*	129	131	129	128	127	128	127	125
* P*-value	0.8283	0.0123	0.5443	0.4120	0.3542	0.9782	0.9680	0.0819
CRP (mg/l)								
Treatment	N/A	70 (39.5–95.5)	174 (135.5–224.5)	182 (128–236.5)	174 (104–228)	145 (88.8–213.3)	131.5 (80.5–207.3)	143 (89–211)
*n*		217	197	194	206	202	198	185
Control	N/A	58 (34.5–88.5)	147 (97.9–204)	145 (91–221.5)	130 (82.1–237.5)	113 (75.5–195)	94 (55.5–161)	92.9 (46.2–147.5)
*n*		126	116	134	125	133	133	121
* P*-value		0.0989	0.0015	0.0006	0.0788	0.0392	0.0010	<0.0001
IL-6 (pg/ml)								
Treatment	165 (86–422)	458 (209–1276)	261 (131–702)	175 (93–413)	130 (55–243)	133 (57–249)	116 (68–224)	79 (50–120)
*n*	96	91	82	78	80	67	74	69
Control	67 (28–144)	103 (58–284)	78 (40–240)	93 (43–258)	61 (31–122)	65 (30–134)	44 (25–82)	39 (21–77)
*n*	103	61	51	50	52	49	57	47
* P*-value	<0.0001	<0.0001	<0.0001	0.0012	0.0010	0.0005	<0.0001	0.0003
PCT (ng/ml)								
Treatment	N/A	2.2 (0.7–6)	2 (0.7–7.8)	1.3 (0.5–6)	1 (0.1–3)	0.7 (0.4–2.3)	0.5 (0.2–2)	0.5 (0.1–1.3)
*n*		50	63	75	93	90	83	95
Control	N/A	0.5 (0.2–1.5)	0.4 (0.1–1.8)	0.3 (0.2–1.3)	0.3 (0.2–1.3)	0.3 (0.1–0.7)	0.2 (0.1–0.5)	0.2 (0.1–0.4)
*n*		53	60	53	62	62	65	66
* P*-value		<0.0001	<0.0001	0.0001	0.0607	<0.0001	<0.0001	0.0006

*aPTT* activated partial thromboplastin time, *AT*-III antithrombin III, *CRP* C-reactive protein, *ER* emergency room, *IL*-6 interleukin 6, *N*/*A* not available, *PCT* procalcitonin, *PTI* prothrombin time index

*P* < 0.05 was considered significant

Data presented as median (interquartile range); *n* = available values

### Plasma fibrinogen levels

At admission to the ER, median FIB levels were lower in the treatment group compared with the control group and remained lower both at day 1 and day 2 post-trauma. From day 3 to day 7, median FIB levels rose above the upper normal range of 4.5 g/l and did not differ between the two groups.

### Other coagulation tests

PTI was lower in the treatment group than in the control group at all timepoints. Compared with the control group, aPTT was prolonged in the treatment group at all timepoints. AT-III was at all timepoints lower in the treatment group in contrast to the control group.

### Haematological and inflammatory parameters

At all timepoints, platelet counts were lower in the treatment group compared with the control group. On admission to the ER, haemoglobin levels were ~17 % lower in the treatment group than in the control group. Throughout all following days post-trauma, levels fell to a stable plateau phase in both groups with slightly lower values in the treatment group at all timepoints.

WBC was above normal (>10 × 10^9^/l) on admission but normal throughout the following days without relevant differences between groups.

CRP, IL-6 and PCT were generally higher in the treatment group at most timepoints. CRP and PCT values were not available on admission. There was a ~2.5-fold increase in CRP levels between day 1 and day 2 followed by a steady increase in levels that peaked at day 3 in the treatment group and day 2 in the control group. Levels then declined up to day 7, but were still ~2-fold higher than the levels recorded on day 1. IL-6 and PCT peak values were seen on day 1 in both groups.

### Subgroup analyses

**Fig. 1:**
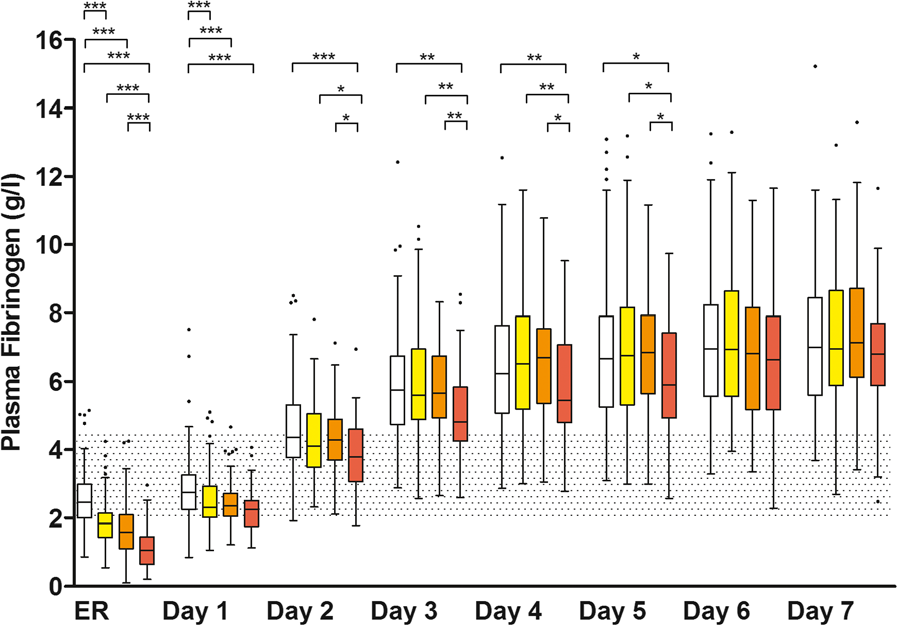
Time course of plasma fibrinogen during the first 7 days after trauma categorized according to the dose of fibrinogen concentrate administered. Control (*white*), 1–4 g FC (*yellow*), 5–9 g FC (*orange*), ≥10 g FC (*red*). Data shown are median, interquartile range and range. Shaded areas represent normal laboratory value ranges (FIB: 2.0–4.5 g/l) and dots represent outliers. ER, values on admission to the emergency room; not indicated = not significant; *P < 0.05; **P < 0.01; ***P < 0.001; in the Newman-Keuls multiple comparisons between all four groups

**Fig. 2:**
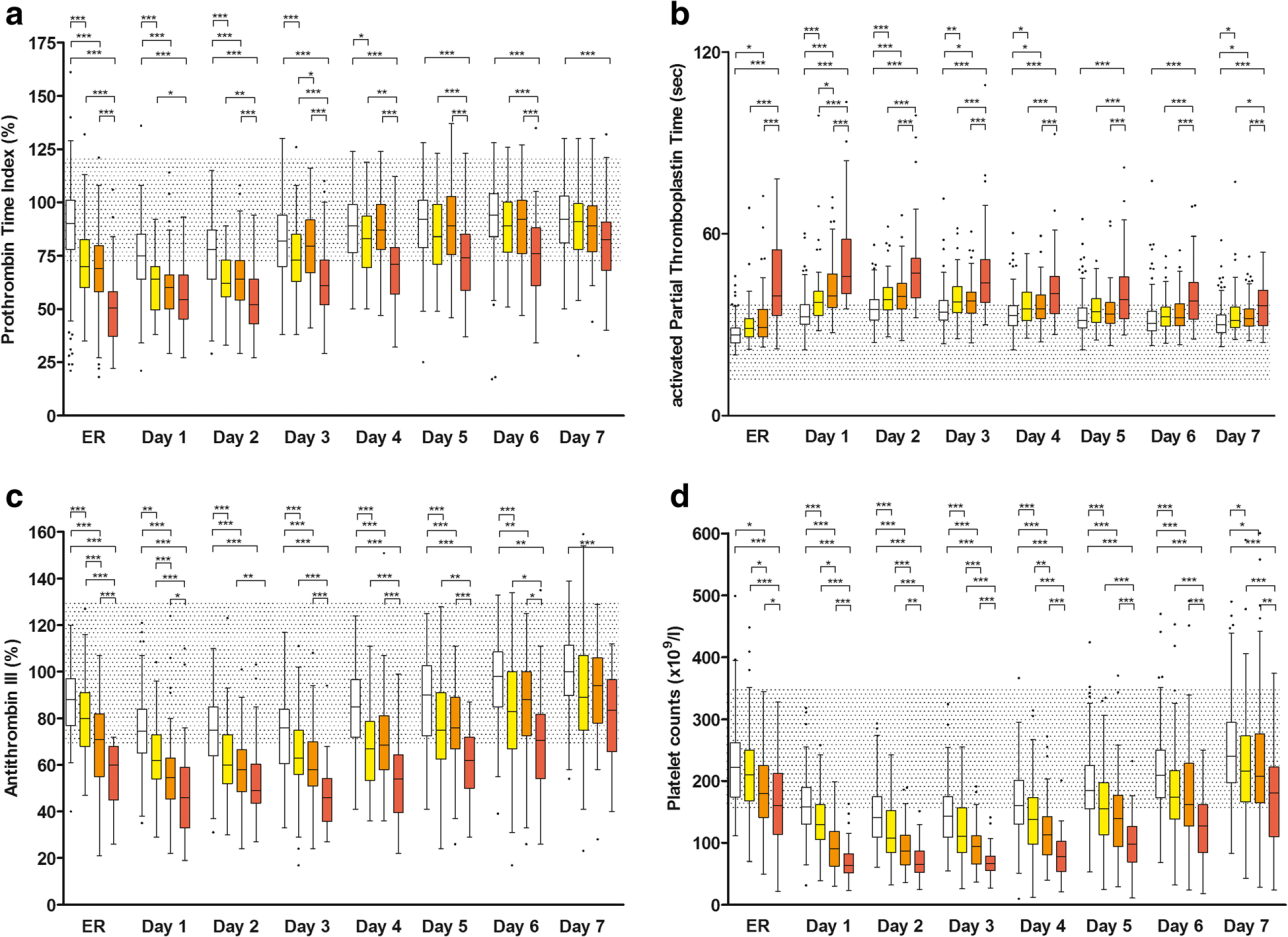
Time course of prothrombin time index (**a**) activated partial thromboplastin time (**b**) antithrombin III (**c**) and platelet count (**d**) during the first 7 days after trauma categorized according to the dose of fibrinogen concentrate administered. Control (*white*), 1–4 g FC (*yellow*), 5–9 g FC (*orange*), ≥10 g FC (*red*). Data shown are median, interquartile range and range. Shaded areas represent normal laboratory value ranges (PTI: 70–120 %; aPTT: 26–35 s; AT-III: 75–125 %; Plt 150–350 × 10^9^/l) and dots represent outliers. ER, values on admission to the emergency room; not indicated = not significant; *P < 0.05; **P < 0.01; ***P < 0.001; in the Newman-Keuls multiple comparisons between all four groups

**Fig. 3:**
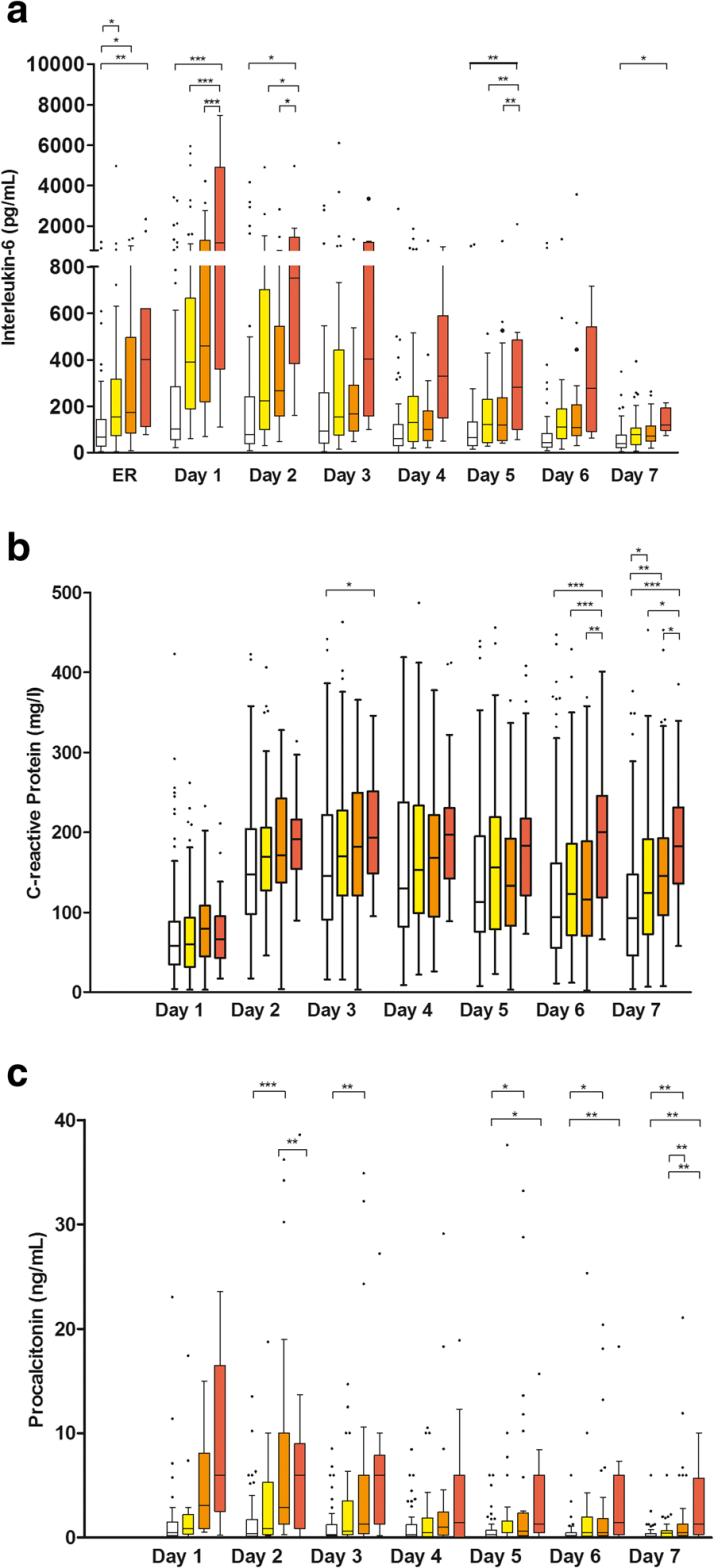
Time course of interleukin-6 (**a**) C-reactive protein (**b**) and procalcitonin (**c**) during the first 7 days after trauma categorized according to the dose of fibrinogen concentrate administered. Control (*white*), 1–4 g FC (*yellow*), 5–9 g FC (*orange*), ≥10 g FC (*red*). Data shown are median, interquartile range and range. Dots represent outliers. ER, values on admission to the emergency room; not indicated = not significant; *P < 0.05; **P < 0.01; ***P < 0.001; in the Newman-Keuls multiple comparisons between all four groups. C- reactive protein and procalcitonin were not measured on admission to the ER

The time courses of FIB, as well as PTI, aPTT, AT-III, platelet count, haemoglobin, CRP and IL-6 during the first 7 days after trauma, categorized according to the dose of FC administered, are shown in Figs. 1, 2 and 3, respectively.

### Plasma fibrinogen levels of subgroups

At admission to the ER and on day 1, FIB levels were lower in each of the three FC subgroups (1–4 g, 5–9 g and ≥10 g FC) compared with the control group (Fig. 1). Thereafter, FIB levels remained lower only in the ≥10 g FC subgroup up to day 5 compared with the control group. At no timepoint was there a significant difference between the 1–4 g and 5–9 g FC subgroups. At admission and from day 2 up to day 5 the subgroups receiving 1–4 g and 5–9 g FC were generally showing higher FIB levels than the ≥10 g FC subgroup

### Standard coagulation tests of subgroups

PTI was lower in the ≥10 g FC subgroup compared with the control group at all timepoints (Fig. 2a). In the 1–4 g and 5–9 g FC subgroups, PTI was lower than in the control group on admission to the ER and up to days 4 and 2, respectively. Both the 1–4 g and 5–9 g FC subgroups had significantly higher PTI values compared with the ≥10 g FC subgroup at all timepoints up to day 6, except day 1 in the 5–9 g FC subgroup. PTI values between the 1–4 g and 5–9 g FC subgroups were different at days 3 and 4 only.

Compared with the control group, aPTT was prolonged in the ≥10 g FC subgroup at all timepoints (Fig. 2b). In the 1–4 g FC subgroup, aPTT was prolonged compared with the control group on days 1–4 and 7. In the 5–9 g FC subgroup, aPTT was prolonged compared with the control group at admission to the ER and on days 1–4 and 7. The ≥10 g FC subgroup exhibited prolonged aPTT compared with both the 1–4 g and 5–9 g FC subgroups at all timepoints. There were no significant differences in aPTT values between the 1–4 g and 5–9 g FC subgroups except on day 1.

AT-III activity was lower in the ≥10 g FC subgroup at all timepoints (Fig. 2c). In the 1–4 g and 5–9 g FC subgroups, AT-III was lower compared with the control group at all timepoints, except on day 7. The ≥10 g FC subgroup exhibited lower AT-III compared with both the 1–4 g and 5–9 g FC subgroups at most timepoints except on day 7. AT-III differed between the 1–4 g and 5–9 g FC subgroups on admission to the ER and on day 1.

### Haematological parameters of subgroups

At almost all timepoints, platelet counts were different between all four groups; highest in the control group, and lowest in the treatment group receiving ≥10 g FC (Fig. 2d). Haemoglobin was different between all four subgroups on admission to the ER only (control group: 132 [118–142] g/l; 1–4 g FC subgroup: 121 [1015–131] g/l; 5–9 g FC subgroup: 113 [95–129.5] g/l; ≥10 g FC subgroup: 84.5 [69–104] g/l). At all subsequent timepoints haemoglobin remained stable at a plateau of ~94 g/l in the control group and ~90 g/l in all subgroups with only slight statistical differences between subgroups.

### Inflammatory markers of subgroups

IL-6 levels were higher in all FC subgroups compared to control at admission to the ER (Fig. 3a). In the ≥10 g FC subgroup IL-6 was elevated at most timepoints compared to any other subgroup.

At the majority of timepoints, between-group comparisons of CRP levels were similar (Fig. 3b). However, on days 6 and 7 CRP levels in the ≥10 g FC subgroup were higher than in any other group. The control group showed lower CRP levels on day 7 than any FC subgroup.

PCT levels were generally high in the ≥10 g FC subgroup, but due to few available values this was mostly insignificant (Fig. 3c).

## Discussion

The most striking result of the current study is that the evolution of FIB levels from day 3 to day 7 is similar between patients with and without FC as haemostatic therapy. Median FIB levels appeared to reach a plateau at approximately 7 g/l between day 5 and day 7 post-trauma, irrespective of initial fibrinogen supplementation. It is also notable in our study that post-traumatic evolution of FIB levels did not differ between the two groups despite higher ISS, transfusion requirements, allogeneic blood product use, or PCC administration in the treatment group.

It has previously been shown that FIB levels at admission to the ER depend partly on factors such as blood loss/dilution, signs of shock and severity of injury [2]. An analysis of trauma patients, categorized according to whether they received FC alone, FC together with PCC, or FC combined with PCC and FFP, showed that FIB levels 24 h post-trauma (day 1) did not differ between treatment groups [24]. We could recently show in a small number of patients treated with FC and PCC, FC alone, or no coagulation therapy, investigating thrombin generation over the time course of 7 days that FIB levels rose in a similar manner [25]. This finding is in line with data from the Cryostat study which revealed that fibrinogen supplementation with cryoprecipitate had no significant effect on plasma FIB levels in trauma patients from 24 h to 28 days post-treatment [27].

In contrast to the assumption that fibrinogen supplementation may potentially lead to higher FIB levels post-trauma, our current data from 435 patients show for the first time that irrespective of the amount of FC given, FIB levels over the following seven days were not higher in the FC treatment subgroups than in the control group that did not receive FC but were even lower up to day 5 in the group receiving 10 g or more. This finding is in agreement with data from a porcine trauma model which demonstrated that administration of human FC did not down-regulate endogenous fibrinogen synthesis [28].

The rise of FIB levels from day 2 onwards in our study can be attributed to an upregulated fibrinogen synthesis in the liver, occurring in both study groups as part of the acute phase response after tissue injury [29]. Increased FIB levels during the acute phase response has also been shown in patients who received FC as targeted first-line haemostatic therapy in aortic surgery; FC provided a specific, significant, short-lived increase in FIB [23]. This short-lived increase in FIB levels following FC administration has also been shown in patients undergoing cystectomy or cardiopulmonary bypass [7, 43]. In both studies, FIB levels were comparable with the control group 24 h post-operatively. However, the time course of FIB in the subsequent post-operative days was not determined in either study. In the current study FIB levels appeared to reach a plateau between day 5 and day 7 post-trauma (with no significant differences between those days), irrespective of initial fibrinogen supplementation. However, FIB levels after day 7 were not investigated; therefore, we do not know whether (post-operative) FIB levels on day 10, as reported by Solomon et al. [23] in aortic surgery would still be in the plateau or already in the declining phase. It is worth noting that the aforementioned study addresses only two timepoints with an 8-day interval in between. Thus, it is unknown whether FIB levels at day 10 represented a continuing increase, or whether FIB levels might have already peaked between days 2 and 10.

Patients in this study were treated according to a goal-directed algorithm [26] based on quickly available ROTEM parameters, such as FIBTEM clot amplitude at 10 min (A10). On admission to the ER, median FIB levels in the group receiving FC were below 2 g/l. Thus, FIBTEM-based diagnosis of impaired fibrinogen contribution to clotting, as carried out in our institution, appears to be consistent with current internationally recommended plasma level triggers [17, 18] in determining the need for fibrinogen supplementation in trauma patients [5]. The current subgroup analysis shows that admission FIB levels in all patients were significantly related to the amount of FC administered in the first 24 h. Goal-directed therapy with FC has been shown to reduce transfusion requirements in various clinical settings [8, 15, 20, 30–33]. In addition, FC has been shown to significantly diminish bleeding volume in both the clinical setting and in animal models of trauma [8, 34–36].

There is evidence to support the effectiveness of FC in a variety of clinical settings. However, only a few studies have reported on the safety profile of FC [23, 30, 32, 33]. The most compelling evidence for the safety of FC has been provided by a 27-year pharmacosurveillance program, which reported a low incidence of thromboembolic events [37]. A preclinical study of trauma has shown that an increase in thrombin generation after FC administration is short-lived (<6 h) [38]. A clinical study did not find an increase in endogenous thrombin potential between 24 h and 7 days post-treatment with FC [25, 38]. Consistent with these findings, the results of our study suggest that for the trauma population, use of FC does not appear to increase the thrombogenic status above that produced by the acute phase response. Furthermore, polymerized fibrin, also known as antithrombin I, can actually inhibit thrombin generation by sequestering thrombin in the forming clot [39], though the antithrombin effect has yet to be quantified with regard to FIB levels.

As was to be expected, haemoglobin was lower at admission in the treatment group at all timepoints and in line with the higher transfusion rate of RBCs in the treatment group. The time course of haemoglobin levels over seven days post-trauma differed slightly between control and treatment groups but mainly remained stable at around 95 and 90 g/l, respectively. This is most likely attributable to a balance of minor blood loss (drains, blood sampling, re-operations) and minor transfusion requirements for RBCs at the ICU, not further investigated for the purpose of this study.

Interestingly, platelet count remained lower in the treatment group at all timepoints and was even more significantly diminished in the subgroups receiving more FC. This may be attributable to the severity of injury and possible higher systemic inflammatory response [40]. This can also be supported by the results of IL-6, CRP and PCT, being higher in the treatment groups in our study. Generally, CRP levels reached maximum levels by day 3 or day 4, which is in agreement with previous studies showing that CRP levels were elevated for several days following trauma and were not correlated with trauma severity [41, 42].

It is of particular interest that in the treatment group PTI was lower throughout and aPTT was prolonged at the majority of timepoints despite the use of PCC in about half of these patients, suggesting these patients have a lower capacity to initiate clotting. It is important to note that a lower PTI or prolonged aPTT may occur despite the higher endogenous thrombin potential that usually exists during haemodilution following trauma and after PCC administration [44, 45]. For example, Dunbar and Chandler showed that when all coagulation factors and inhibitors in plasma are diluted, thrombin generation increased and peaked when the plasma was diluted to 40 % of normal [44].

Interestingly, one patient in the control group received no FC therapy despite receiving massive transfusion. This patient, who was bleeding and on pre-trauma vitamin K antagonist therapy, needed PCC only, and maintained sufficient functional fibrin polymerisation throughout RBC transfusion.

Our study has several limitations. Data were collected retrospectively from a single centre, and the baseline characteristics between the two groups were not comparable. We included only those patients with FIB values available post-trauma up to day 6 or 7. For the purpose of this study we documented FC, PCC, platelet concentrate and RBC use in the first 24 h only. Therefore, the potential effects of similar therapies after 24 h on the study parameters could not be determined–nor could the potential impact of other therapies at any timepoint be excluded (e.g., tranexamic acid). The data in this study were collected over a long time period (2005–2015), during which changes occurred in our clinic’s standard transfusion protocols e.g., initially, PCC was administered more liberally but now this is only administered after FC administration and an ongoing prolonged EXTEM clotting time (CT). Therefore, differences in patient management might have impacted our assessments of patients’ haemostatic outcomes. We cannot exclude the impact of age on the evolution of FIB levels; interestingly, patients in the control group were significantly older than those in the treatment group. However, in the range of observed ages we consider this difference to be of minor importance. It is also noteworthy that some patients in the control group who did not present with bleeding at admission did not receive FC despite FIB levels below the current recommended threshold levels. We did not investigate the prothrombotic effects of FC in this study and were unable to include any information on thromboembolic events as patients are not routinely screened for deep vein thrombosis in our centre. The main problem being that markers of thromboembolic events, such as D-dimers, are not suitable for establishing these events in trauma patients. However, due to the overall equal time course of FIB levels regardless of administration of FC, this should not impact such events per se.

Furthermore, the tendency for lower fibrinogen in the highest FC group may be because of the need for further operations after the initial damage control surgery in those patients with more severe trauma. We cannot exclude that this may have biased the results of this group to some extent. However, it is interesting that the patients who received the highest amount of FC had the most severe injuries and presumably the most secondary “hits” (operations), and still present with a tendency to lower fibrinogen levels.

## Conclusions

In conclusion, treatment of bleeding trauma patients with FC during the first 24 h after hospital admission does not affect the evolution of FIB levels from day 3 to day 7 post-trauma compared with controls. No additional increase in FIB was observed beyond that occurring naturally due to the acute phase response after tissue injury. We therefore suggest that administration of FC to the trauma patient is unlikely to increase prothrombotic status. Prospective, multicentre studies are needed to confirm our results.

### Key messages

- It has been questioned whether fibrinogen concentrate (FC) therapy has the potential to abnormally increase plasma fibrinogen levels thus leading to a prothrombotic risk following trauma
- In this retrospective study, trauma patients receiving FC therapywere compared with patients not receiving FC
- Patients treated with FC had higher injury severity scores and higher transfusion of allogeneic blood products than those not receiving FC, butplasma fibrinogen levels from day 3 to day 7 post-trauma were similar in the two patient groups, reaching a plateau of ~7 g/l between day 5 and day 7
- These data show that FC therapy does not lead to higher plasma fibrinogen levels among trauma patients

## Abbreviations

A10: FIBTEM clot amplitude at 10 min
aPTT: activated partial thromboplastin time
AT-III: antithrombin III
CRP: C-reactive protein
ER: emergency room
FC: fibrinogen concentrate
FFP: fresh frozen plasma
FIB: plasma fibrinogen
ICU: intensive care unit
IL-6: interleukin 6
IQR: interquartile range
ISS: injury severity score
PCC: prothrombin complex concentrate
PCT: procalcitonin
PTI: prothrombin time index
RBC: red blood cells
WBC: white blood cell count
